# Primitive Auditory Memory Is Correlated with Spatial Unmasking That Is Based on Direct-Reflection Integration

**DOI:** 10.1371/journal.pone.0063106

**Published:** 2013-04-29

**Authors:** Huahui Li, Lingzhi Kong, Xihong Wu, Liang Li

**Affiliations:** 1 Department of Psychology, Peking University, Beijing, China; 2 Department of Machine Intelligence, Peking University, Beijing, China; 3 Speech and Hearing Research Center, Peking University, Beijing, China; 4 Key Laboratory on Machine Perception (Ministry of Education), Peking University, Beijing, China; 5 PKU-IDG/McGovern Institute for Brain Research, Peking University, Beijing, China; Hotchkiss Brain Institute, University of Calgary, Canada

## Abstract

In reverberant rooms with multiple-people talking, spatial separation between speech sources improves recognition of attended speech, even though both the head-shadowing and interaural-interaction unmasking cues are limited by numerous reflections. It is the perceptual integration between the direct wave and its reflections that bridges the direct-reflection temporal gaps and results in the spatial unmasking under reverberant conditions. This study further investigated (1) the temporal dynamic of the direct-reflection-integration-based spatial unmasking as a function of the reflection delay, and (2) whether this temporal dynamic is correlated with the listeners’ auditory ability to temporally retain raw acoustic signals (i.e., the fast decaying primitive auditory memory, PAM). The results showed that recognition of the target speech against the speech-masker background is a descending exponential function of the delay of the simulated target reflection. In addition, the temporal extent of PAM is frequency dependent and markedly longer than that for perceptual fusion. More importantly, the temporal dynamic of the speech-recognition function is significantly correlated with the temporal extent of the PAM of low-frequency raw signals. Thus, we propose that a chain process, which links the earlier-stage PAM with the later-stage correlation computation, perceptual integration, and attention facilitation, plays a role in spatially unmasking target speech under reverberant conditions.

## Introduction

### Perceptual Integration and the Psychological Unmasking Effect of Spatial Separation

Listeners with normal hearing are able to recognize the attended speech under noisy, multiple-people-talking conditions. “How do we recognize what one person is saying when others are speaking at the same time?” This *cocktail-party problem*, first proposed by Cherry, has puzzled people for half a century [1]. It reflects the humans’ remarkable ability to use various spatial and/or non-spatial cues to facilitate selective attention to target speech and follow the target stream against irrelevant-speech influences (for a recent review see [2]).

Spatial separation between a target sound and its masking sounds improves recognition of the target signal by a number of unmasking effects, including (1) the acoustic effect of head shadowing that increases the signal-to-masker ratio (SMR) in sound-pressure level at the ear near the target, (2) the neurophysiological effect of the disparity in arriving-time difference between inputs to the two ears (i.e., the interaural-interaction effect), and (3) the psychological effect of selective attention that facilitates the target salience and reduces the masker salience. Interestingly, when the listening environment is reverberant (such as in a cave or room with hard surfaces), although numerous reflections bouncing from surfaces limit or even abolish both the head-shadowing and the interaural-interaction unmasking effects, the psychological unmasking is still effective (e.g., [3]–[8]). Why does the psychological effect persist under reverberant conditions?

Caves had long been essential for human ancestors to survive. Archaeological evidence shows that activities of prehistoric cave dwellers (as indicated by palaeolithic cave paintings) were associated with cave reverberation [9], suggesting that the auditory system of human ancestors confronted the natural pressure to deal with reflections in every-day living conditions. Not surprisingly, humans exhibit the extraordinary ability to perceptually integrate the direct sound wave of a source with its time-delayed and linearly filtered reflections: Attributes of the reflections can be perceptually captured by the direct wave [10], even resulting in a single fused image of the source whose perceived location is around the location of the source (the precedence effect, [11]–[15]). The precedence effect plays a role in suppressing the perception of distinct *echoes* and facilitating the recognition and localization of sources in reverberant environments.

Compared to non-speech sounds such as clicks and noise bursts, speech sounds have a much higher perceptual fusion tendency (i.e., larger echo threshold, [11], [15]–[18]). In a reverberant environment, to perceptually segregate a target signal from other disruptive stimuli (which will not be as highly correlated with the target signal), the auditory system must not only integrate sound waves that directly come from the signal source with reflections of the signal source, but also at the same time, integrate sound waves that come from a disruptive source with reflections of the disruptive source. Otherwise the auditory scene will be cluttered and confusing. Thus, the high perceptual fusion tendency for speech sounds is beneficial for speech recognition under the adverse listening condition.

Indeed, since the pioneering study by Freyman and his colleagues [4], it has been well confirmed that the precedence-effect-related integration of speech sounds plays an essential role in improving speech perception under simulated reverberant and multiple-people-talking conditions by inducing perceived spatial separation between the target speech and the masking speech [4], [19]–[23]. For example, based on the principle of the precedence effect (the coherent sound waves delivered from two spatially separated loudspeakers are perceptually fused), when both the target speech and the masker (either speech masker or noise masker) are presented by a loudspeaker to the listener’s left and another loudspeaker to the listener’s right, the perceived location of the target and that of the masker can be manipulated by changing the delay between the two loudspeakers for the target signals and the masker signals (for details see [19]). Moreover, if the masker is speech, recognizing target speech under the condition of perceived target-masker spatial separation is markedly better than that under the condition of perceived target-masker co-location, even though neither the masker energy at each ear nor the masker-image compactness/diffusiveness is substantially changed. However, when the masker is steady-state speech-spectrum noise, such a spatial separation leads to a relatively smaller (but significant) release. Because steady-state speech-spectrum noise only produces energetic masking and a speech masker produces both energetic masking and informational masking (for the concepts of energetic masking and informational masking see [4], [25]–[29]), it appears that perceptual segregation between target speech and masking speech mainly reduces informational masking of target speech. The reduction of informational masking is caused by the enhanced perceptual differences (i.e., in perceived spatial location) between target speech and masking speech, leading to improved selective attention to target speech [30].

The advantage of perceptual integration in unmasking speech can occur over a large range of lead-lag intervals. For example, in the Rakerd et al. study, a two-talker speech masker was presented by two spatially separated loudspeakers [22]. One loudspeaker was located directly in front (at 0°) and the other one was 60° to the right of the listener. The inter-loudspeaker time interval for the speech masker (inter-masker interval, IMI) was varied in a broad range from −64 to +64 ms. At the same time the target speech was presented only by the frontal loudspeaker. The results showed that when the absolute value of IMI was 32 ms or shorter, there was consistent evidence of release from speech masking for target-speech recognition. However, when the IMI was either −64 or +64 ms, there was no evidence of release from masking. If the masker was speech-spectrum noise, significant release occurred only at a few short IMI less than 4 ms. Thus, the release of target speech from speech masking over a range of IMIs between 4 and 32 ms cannot be explained by a reduction in energetic masking, and perceptual integration of the leading and lagging speech maskers must play a role in reducing informational masking of target speech. Moreover, for the masker signals, even when the loudspeaker that delivered both the target and the masker led the loudspeaker that only delivered the masker by a time interval between 0 and 32 ms (when there was no perceived spatial separation between the target and the masker), the release was still evident, suggesting that in addition to introducing differences in perceived spatial location, introducing differences in auditory image (compactness/diffusiveness, timbre, and/or loudness) between target speech and masking speech can unmask target speech.

Obviously, to parse the auditory scene in a noisy, reverberant environment, perceptual integration occurs not only between correlated masking stimuli but also between the direct sound wave coming from the target source and the target reflections. Since listeners normally try to attend to target signals and ignore masking stimuli, the function of perceptually integrating target stimuli is as important as that for masking stimuli. In our recent studies [23], [31], to investigate the unmasking function of perceptual integration of target speech and simulated target-speech reflection, the strength of the perceptual integration of target speech signals was modulated by changing the time interval between the target speech and its spatially-separated single-reflection simulation (inter-target interval, ITI) over the range between 0 and 64 ms. The results showed that reducing the ITI from 64 to 0 ms not only progressively enhanced perceptual integration of target-speech signals, but also progressively released target speech from masking, especially from speech masking (the release from speech masking was larger than the release from noise masking). The target-reflection integration must cause certain perceptual differences (e.g., in spatial location, compactness, and/or loudness) between the target image and the background-masker image to help participants selectively attend to the target signals, leading to a release of the target speech from the masker.

### Primitive Auditory Memory

As mentioned above, the strength of perceptual integration depends on the reflection delay time: Increasing the delay reduces both perceptual fusion [31] and target-speech recognition against speech masking [23], [31]. Surprisingly, this function of the reflection delay varies considerably across normal-hearing younger adults [23]. The dependence of perceptual integration on the reflection delay implies that a temporal storage of raw signals of the direct (leading) wave is necessary. In fact, since auditory information is processed in a temporally sequential pattern, both an auditory storage and a readout of sequential auditory information from the storage are critical for organizing acoustic stimuli into auditory-image units [32]. Theoretically, without a faithful storage of raw signals of the leading wave, neither the central computation of the similarity (correlation) nor the perceptual integration between the leading and lagging waves is possible. This faithful auditory storage of raw signals (such as fine structures of wideband noises) has been termed *primitive auditory memory* (PAM) and recognized as the early point in the chain of the transient auditory memory system [23], [24]. How to examine the listener’s ability to temporally store acoustic raw signals (such as fine structures) of a sound source (such as a random noise)?

**Figure 1:**
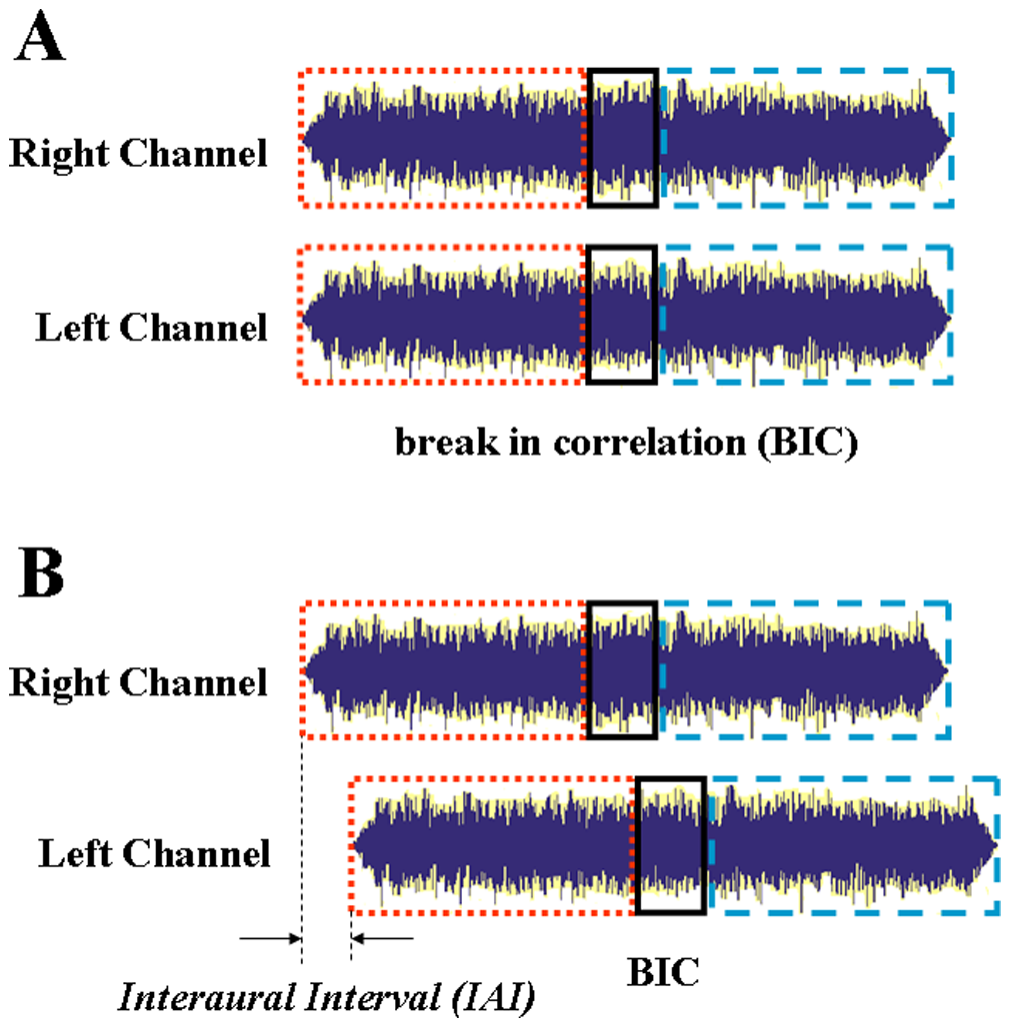
Illustration of the concept of *break in correlation* (BIC). *A*: An interaurally uncorrelated noise fragment (i.e., a BIC, as indicated by the solid frame) is inserted in the temporal middle of an interaurally identical steady-state noise (as indicated by dash and dot frames). *B*: An interaural interval (IAI) is introduced on the base of *A* for the measurement of primitive auditory memory (PAM).

Humans are extremely sensitive to differences between a wideband noise delivered at one ear and its copy delivered at the other ear [33]–[37]. Changing the interaural correlation of wideband noises modifies the percept of the noises [38], [39]. For example, when the interaural correlation of wideband pink noises is 1, listeners perceive a single compact auditory event precisely localized in the middle of the head; when the interaural correlation is 0, listeners perceive two respective events, one at each ear; when the interaural correlation is 0.25, 0.50, or 0.75, listeners perceive one diffused event in the median plane and two additional ones lateralized symmetrically with respect to the median plane [38]. Thus, introducing a change in interaural correlation of the arbitrary noises does not alter the energy and spectrum but modifies some perceptual dimensions such as the compactness, number of images, and lateral position. Consequently, human listeners with normal hearing are able to detect a dynamic break in interaural correlation (BIC) (i.e., a brief drop of interaural correlation from 1 to 0 and then return to 1, Figure 1A) in interaurally correlated steady-state noises [23], [24], [35], [36], [40]–[42].

The perceptual representation of interaurally correlated noises is also determined by the delay time between the two ears (i.e., interaural time difference, here called interaural interval, IAI). As mentioned above, if identical steady-state wideband noises are presented at the two ears with the IAI of 0 ms, a single compact noise image is perceived at the middle point inside the head of a normal-hearing listener. When an IAI shorter than 1 ms, e.g., 0.5 ms, is introduced, the image is located between the middle of head and the leading ear. When the IAI is increased to 1 ms, the image is perceived at the leading ear. With a further increase of the IAI to a higher value within the range of the precedence effect, e.g., 4 ms for steady-state speech-spectrum noise, a single fused noise image is still located at the leading ear but the compactness of the noise image is reduced. Yost (1981) estimated the lateral position within the head for binaurally-presented tones with various frequencies and showed that although cross correlation or a coincidence network can account for the results at any one frequency, it cannot account for the results across frequencies. In other words, the image is not at the same position for a particular value of IAI at various frequencies [43]. More specifically, the image position within the head appears to be located at positions closer to midline as the tone frequency increases and the range of the image distributions (which can be used to describe the degree of diffuseness) is also affected by the tone frequency. Thus, introducing an IAI no larger than 1 ms for interaurally-correlated wideband noises may lateralize different frequency components of the auditory event differently (i.e., lower frequency components further away from the midline than higher frequency components). If such a difference in intracranial position occurs across frequencies, the compactness of the noise image may be weakened, thereby reducing the sensitivity to a drop in interaural correlation (possibly due to compressed changes in event size). However, this difference in lateral displacement across frequencies cannot account for the continued increase in diffuseness of the noise percept as the IAI increases to values far beyond the largest ecological IAI.

Theoretically, when an IAI of several milliseconds or longer is introduced, fine-structure information of the steady-state noise at the leading ear has to be maintained in the central auditory system for that period of time, otherwise, instead of one single fused image, multiple images would be perceived. However, when the IAI is sufficiently long (e.g., larger than the fusion threshold), a distinct noise image is perceived at each of the two ears (the perceptual fusion is broken). Interestingly, even when a large IAI (up to 20 ms) is introduced (Figure 1B), the BIC is still detectable [24], [40]–[42] and a faint dichotic repetition pitch is heard [44], indicating that raw signals of the leading-ear noise can be stored during this interval, allowing the central computation of the similarity (correlation) between the binaural inputs. Obviously, detecting the BIC is based on the contrast in interaural correlation between the central representation of the BIC and that of the BIC banks (the noise sections flanking the BIC). As the IAI becomes larger, the central storage of raw signals of the noise entering the leading ear continues to decay and the interaural correlation of the BIC banks (flanking the BIC) in the central representation gradually reduces. Since the interaural correlation for the BIC is always about zero, the decrease in the interaural correlation of the central representation of the BIC banks reduces the contrast in interaural correlation between the BIC banks and the BIC, leading to that listeners feel it more difficult to detect the BIC. Thus, measuring the longest IAI between the two ears, at which a BIC is still detectable, is a way for estimating the temporal extent of the PAM [23]. Note that introducing a change in interaural correlation for wideband noises does not change the energy and spectrum in the signals, but it can change the loudness of the signals [46] and dichotic repetition pitch [44].

### The Present Study

The present study was to investigate whether the perceptual integration-based speech recognition against speech masking, which is vulnerable to the reflection delay, is functionally related to the listener’s auditory ability to maintain fine-structure acoustic signals (i.e., the PAM). The temporal extent of the PAM is operationally defined as the longest IAI at which a BIC with a fixed duration is detectable, and the temporal dynamic of the decay of PAM is estimated by measuring the increase in the duration threshold for detecting the BIC as the IAI increases.

In Experiment 1 of this study, under a simulated reverberant condition with multiple-people talking, the temporal dynamic of the speech-recognition function of the reflection delay was investigated in 30 younger adults with normal hearing. More specifically, the identical target sentences were presented through two spatially separated loudspeakers, which were located symmetrically to the left-front and the right-front of the listener in an anechoic room. The target sentence presented through the right loudspeaker always led the target sentence presented through the left loudspeaker by a time interval, i.e. inter-target interval (ITI). The target sound from the right loudspeaker arrived earlier at the listener’s ears, simulating the direct sound wave from a sound source. The later-arriving target sound from the left loudspeaker simulated the first reflection of the sound source. Thus, this was a highly simplified simulation of reverberation, and the degree of reverberation was manipulated by varying the ITI over the range between 0 and 64 ms. In this experiment, since we focused on the effect of reflection delay of the target speech, there was no simulation of reverberation for speech maskers.

In Experiment 2, the temporal extent of PAM was examined in the same 30 participants who participated in Experiment 1.

In Experiment 3, to examine whether the BIC is still audible at long IAIs where the perceptual fusion of the BIC banks is already broken (i.e., when the noise image is perceived at each of the two ears), both the temporal extent of PAM and the IAI threshold for perceptually fusing the interaurally correlated noise were measured in 13 younger adults with normal hearing.

In Experiment 4, since investigation of the temporal dynamic feature of PAM is critical for understanding the nature of PAM, this dynamic feature was estimated by measuring the change in the BIC-duration threshold for detecting the BIC as the IAI was varied between 0 and 10 ms. If there is a degeneration of the interaural integration of fine-structure details by introducing an IAI, a BIC should be less detectable, leading to an increase in the duration threshold for detecting the BIC. Thus, measuring the duration threshold for detecting the BIC at various IAIs provides a way of investigating whether the interaural integration of acoustic details is affected by the interaural delay.

Finally, correlations were calculated between the temporal dynamic of the speech recognition as a function of the reflection delay (obtained from Experiment 1) and the longest IAI for both wideband and narrowband noises (obtained from Experiment 2) across 30 participants.

## Experiment 1: Speech Recognition under the Simulated Reverberation Condition with Speech Masking

### Ethics Statement

In this and the following experiments of this study, all participants provided informed written consent to participate. Both the consent procedure and the experimental procedures of this study involving human participants were approved by the Committee for Protecting Human and Animal Subjects in the Department of Psychology at Peking University.

### Participants

Thirty university students (5 males and 25 females; mean age = 22.5 yrs between 19 to 27 yrs) provided informed consent and participated in both Experiment 1 and Experiment 2. They all had symmetrical hearing (no more than 15-dB difference between the two ears) and normal pure-tone hearing thresholds (no more than 25 dB HL) between 0.125 and 8 kHz (ANSI-S3.6, 2004). They were paid a modest stipend for their participation.

### Apparatus and Stimuli

**Figure 2:**
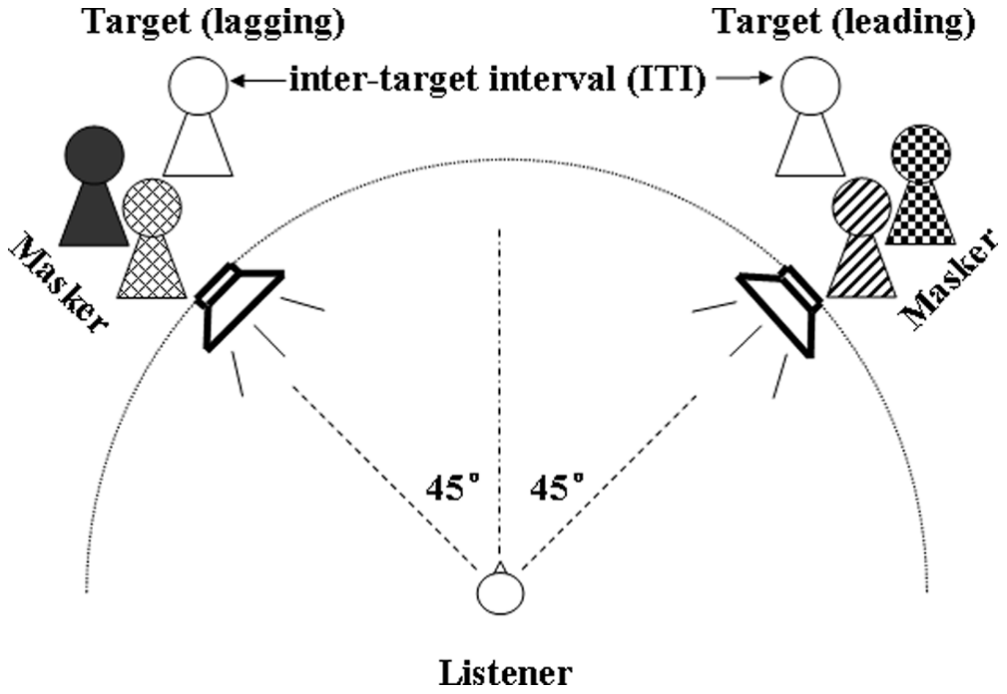
Illustration of the spatial locations of the two loudspeakers relative to the listeners in Experiment 1. Sound signals were delivered to two loudspeakers symmetrically placed to the left and right 45° of the listener. The target speech (indicated by the unfilled human-shape symbol) was presented by the left and the right loudspeakers as the right one led the left one by an inter-target interval (ITI). Speech maskers spoken by four female talkers (indicated by the filled human-shape symbols) were presented to the loudspeakers, two on the left and two on the right.

The participant was seated in a chair at the center of an anechoic chamber (Beijing CA Acoustics), which was 560 cm in length, 400 cm in width, and 193 cm in height. All acoustic signals were digitized at the sampling rate of 22.05 kHz. The acoustic analog outputs were delivered to two loudspeakers in the frontal azimuthal plane at the left and right 45° positions with respect to the median plane (Figure 2). The loudspeaker height was 106 cm, approximately ear level for a seated listener with average body height. The distance between the loudspeaker and the center of the participant’s head was 200 cm.

Speech stimuli are Chinese “nonsense” sentences, which are syntactically correct but not semantically meaningful. Direct English translations of the sentences are similar but not identical to the English nonsense sentences used in previous studies (e.g., [4], [19]). Each sentence has 12 characters (also 12 syllables) including three key components (also the three keywords): subject, predicate (verb or copula), and object, with two characters (also two syllables) for each keyword. For example, the English translation of one Chinese nonsense sentence is “*This polyester will expel that stomach*” (the keywords are underlined). Note that the sentence structure cannot provide any contextual support for recognizing the keywords. The development of the Chinese nonsense sentences was described elsewhere [29].

Target speech was spoken by a young female talker (Talker A). The speech masker presented from the left loudspeaker was a 47-s loop of digitally-combined continuous recording of Chinese nonsense sentences spoken by two young female talkers (Talkers B and C) different from the target speaker (Figure 2). Also, the speech masker presented from the right loudspeaker was a 47-s loop of digitally-combined continuous recording of Chinese nonsense sentences, spoken by another two young female talkers (Talkers D and E) (Figure 2). Each of the four masking talkers spoke different sentences (whose keywords did not appear in any of the target sentences), and the sound pressure levels were the same across the four masking talkers’ speech sounds. Since both the content and voices of masking speech at one loudspeaker were different from those at the other loudspeaker (Figure 2), there was no perceptual integration between the uncorrelated masking stimuli presented from the two loudspeakers. Also, since the masker started from a different point in the loop for each trial, the left-loudspeaker loop was presented asynchronously with the right-loudspeaker loop on a trial-by-trial basis.

The sound pressure level of the target and masker sounds presented from a loudspeaker (when it was playing alone) were adjusted to 58 dBA and 66 dBA, respectively, producing an SMR of −8 dB.

### Design and Procedures

The two loudspeakers presented identical target sentences with the right loudspeaker leading the left loudspeaker by 0, 16, 32, 48 or 64 ms, while different two-talker speech maskers were presented through both loudspeakers at the same time (Figure 2). The order of ITI was counterbalanced across participants. A list of eighteen target sentences was used for each condition. To balance information quantity across stimulus conditions, the information quantity of a keyword in a sentence was calculated as.
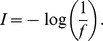
where *f* is Chinese-word frequency, which is based on the database of the Chinese newspaper *People’s Daily* published over 9 years (1994–2002). Information quantity of a sentence was the sum of information quantities of the three keywords. Sentence lists were constructed in a way that the information quantity of each sentence list (for each ITI condition) was about the same [29].

In each trial, the participant pressed a button on the response box to start the speech masker. About 1 second later, a target sentence was presented along with the masker, and the masker and target ended simultaneously. Participants were instructed to loudly repeat the whole target sentence immediately after all the stimuli ended. Performance was scored on the number of correctly identified syllables for each keyword.

To ensure that participants fully understood and correctly followed the experimental instructions, in this and the following experiments a short training session was conducted before formal testing.

## Results and Discussion

**Figure 3:**
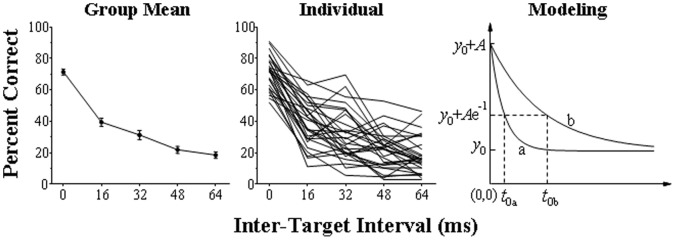
Percent-correct recognition of target speech as a function of the ITI (Experiment 1). The group-mean and individual percent-correct recognition of target speech at various ITIs are showed in *left* and *middle panels*, respectively. The error bars represent the standard errors of the mean. *Right panel*: Two modeling curves of target-speech recognition are the exponential function of the ITI. Compared to curve a, curve b has a larger time constant (*t*_0_), indicating a slower decline of the exponential function of the ITI for curve b.

In Experiment 1, using a simulated reverberant environment with multiple-people talking, the temporal dynamic of the speech-recognition function of the reflection delay was measured and mathematically modeled in 30 participants. Similar to previous studies [31], in this experiment when the ITI was 0 ms, the perceptual integration of the target speech was the strongest: One compactly fused target image was perceived as coming from the frontal field. When the ITI was 16 ms, participants perceived one less fused target image as coming from the semi-field with the leading (right) loudspeaker. With further increasing the ITI towards 64 ms, two distinct target images were perceived, near left and right loudspeakers, respectively. Note that changing the ITI does not alter the long-term average power of the target-speech signal [31]. As shown in the left panel of Figure 3, the group-mean percent-correct recognition of target speech significantly declined as the ITI increased from 0 to 64 ms [one-way ANOVA: F(4,116) = 158.935, *p*<0.001].

The middle panel of Figure 3 shows the percent-correct recognition of target speech as a function of ITI for individual participants. To mathematically model this function, each participant’s data were fit with an exponential function:
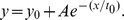
where *y* is the percent-correct speech recognition, *x* is the corresponding ITI, *y*_0_ is the baseline percent-correct recognition not affected by ITI, *A* is the range of performance affected by ITI, and *t*_0_ is the time constant of the exponential function (determining the rate of the performance decline as the ITI increases). Clearly, a larger *t*_0_ represents a slower decline of target recognition performance as the ITI increases. Thus, *t*_0_ represents the temporal dynamic of the speech recognition as a function of the ITI, determined by the perceptual integration between the direct and reflection target waves. To illustrate the effect of *t*_0_, the right panel of Figure 3 shows two modeling curves of speech recognition against ITI, with the same *y*_0_ and *A* but different *t*_0_s (the *t*_0b_ for curve b is larger than the *t*_0a_ for curve a).

**Figure 4:**
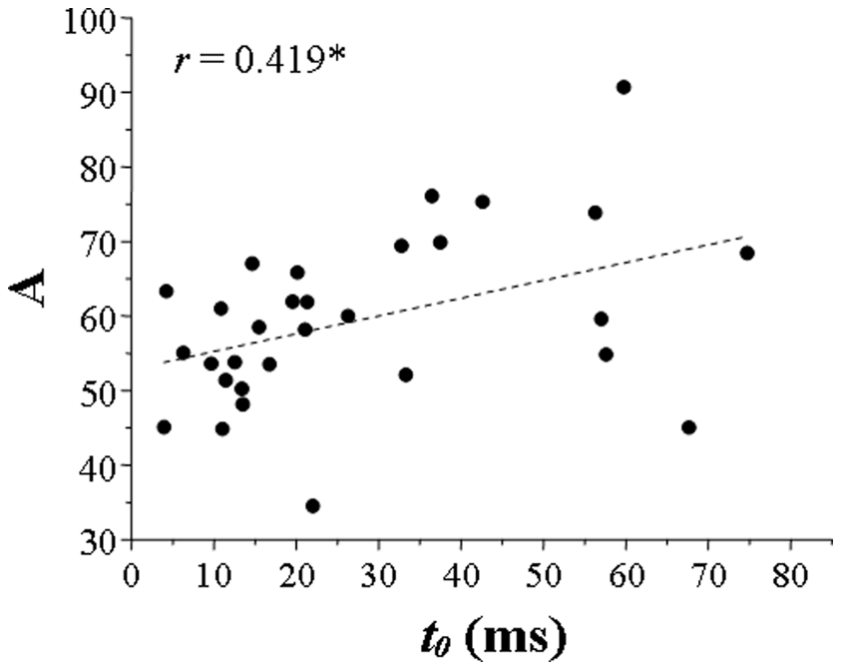
Correlation between the parameter *A* (the dynamic range of the performance change affected by ITI, the value along the ordinate) and the time constant *t*_0_ (the value along the abscissa) across participants (Experiment 1). The dashed line is the best linear fitting for the data points, and *r* is the Pearson correlation coefficient. ‘*’ indicates significance at the level of 0.05.

Then, the resulted *t*_0_s for 30 individual participants are shown as the abscissa values in Figure 4. Obviously, a large variability in *t*_0_ occurred across participants. Figure 4 also shows individual participants’ *A*s (values along the ordinate), indicating a considerable inter-participant variability in the range of performance affected by ITI. A weak but significant correlation occurred between *A* and *t*_0_.

## Experiment 2: The Longest IAI for Detecting the BIC

In Experiment 2, the temporal extent of PAM was examined by measuring the longest IAI for detecting the BIC in the same 30 participants who participated in Experiment 1.

### Participants

The 30 participants who participated in Experiment 1 participated in Experiment 2.

### Apparatus and Stimuli

The participant was seated in a sound-attenuating chamber (EMI Shielded Audiometric Examination Acoustic Suite). Gaussian wideband noise signals were synthesized using the “randn()” function in the MATLAB function library at the sampling rate of 48 kHz with 16-bit amplitude quantization, which was then low-pass filtered at 10 kHz for wideband-noise stimulation condition or band-pass filtered with a bandwidth of 1/3 octave and a center frequency (CF) of 200, 400, 800, 1600 or 3200 Hz for narrowband-noise stimulation conditions. The spectral shaping had linear band edges of 37.5, 75, 150, 300, or 600 Hz for the five narrowband noises, respectively. Each noise stimulus has the duration of 2000 ms including 30-ms rise-fall times. All stimuli were transferred using the Creative Sound Blaster PCI128, passed through an AURICAL system, and presented to listeners through headphones (Model HDA 200). Calibration of sound level was carried out with the Larson Davis Audiometer Calibration and Electroacoustic Testing System (AUDit and System 824, Larson Davis, Depew, NY). The sound pressure level at each headphone was fixed at 58 dBA.

### Design and Procedures

Each trial included two binaural presentations of 2000-ms noises. In one presentation, the left-headphone noise was an exact copy of the right-headphone noise. In the other presentation, the left-headphone noise was also identical to the right-headphone noise except that its temporal middle was substituted with a randomly selected independent noise fragment (i.e., the BIC) with a fixed duration of 200 ms before filtering (Figure 1). In each trial, the BIC had equal possibility to be randomly assigned to one of the two presentations. The offset-to-onset interval between the two presentations was 1000 ms. For each presentation, the noise presented through the right headphone always started simultaneously with or led that presented through the left headphone, and the IAI was systematically manipulated. Fresh noises were used for each trial. The participant’s task was to identify which of the two presentations contained the BIC. The order of presented noise type was counterbalanced across participants.

The participant initiated a trial by pressing the left button of a mouse. The longest IAI for BIC detection was measured using a three-up-one-down paradigm [47]: the IAI started from 0 ms, increased following three consecutive correct identifications of the presentation containing the BIC, and decreased following one incorrect identification. The initial step size of changing the IAI was 16 ms, which was altered by a factor of 0.5 with each reversal of direction until the minimum size of 1 ms was reached. Visual feedback was given after each trial to indicate whether the identification is correct. A test session was terminated following 10 reversals in direction, and the longest IAI for a session was defined as the mean IAI for the last 6 reversals. The average over the 3 best measures out of 4 repeated test sessions was used as the longest IAI for each participant.

### Results and Discussion

**Figure 5:**
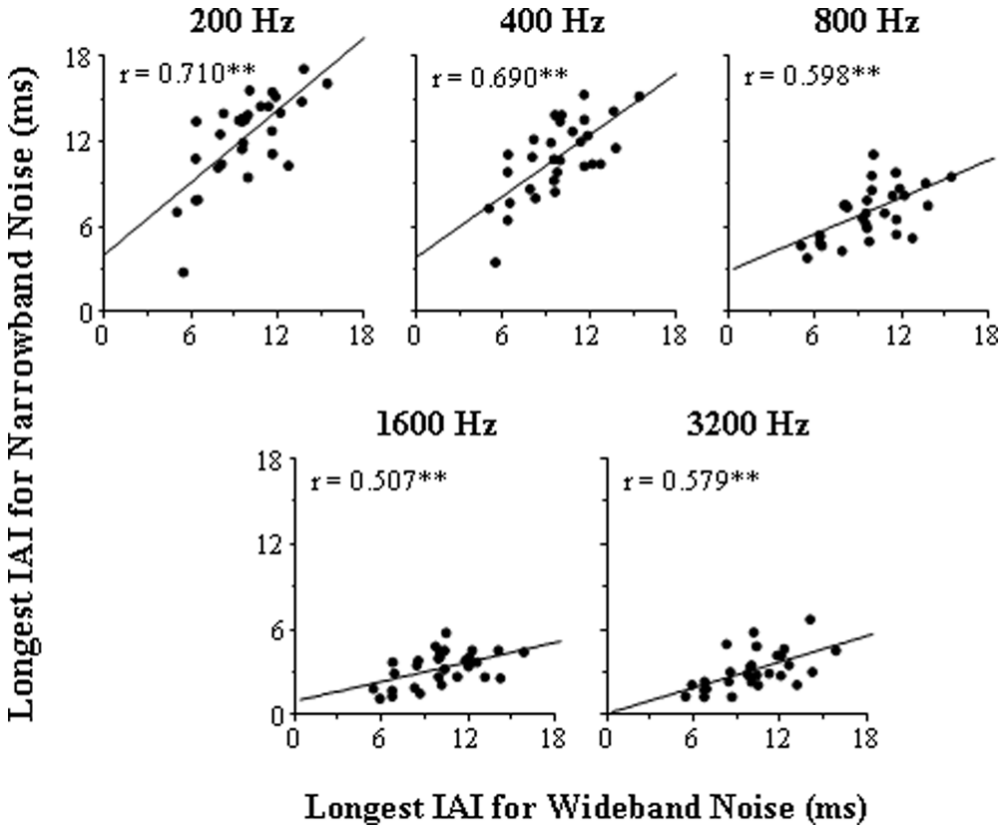
Correlation between the longest interaural interval (IAI) at which a 200-ms break in correlation (BIC) could be detected for each of the five types of narrowband noises and that for wideband noise (Experiment 2). The central frequency (CF) of the narrowband noise was 200, 400, 800, 1600, or 3200 Hz. The solid line in each panel is the best linear fitting for the data points, and *r* is the Pearson correlation coefficient. ‘**’ indicates significance at the level of 0.01.

**Figure 6:**
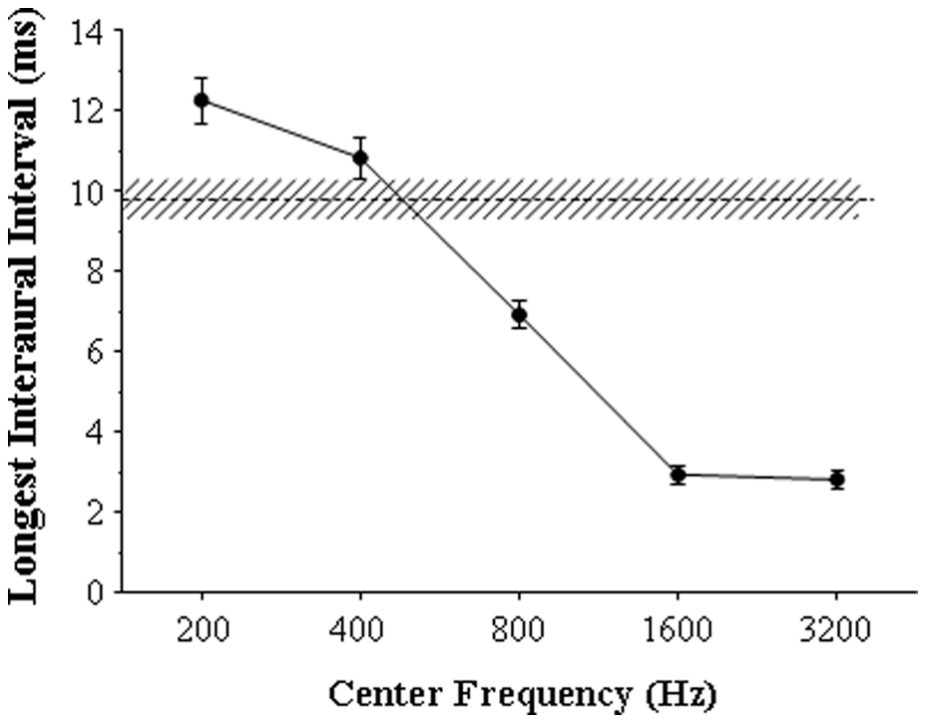
The group mean of the longest IAI for detecting the BIC in the wideband noise and that in each of the narrowband noises (Experiment 2). The dashed line and the filled circles represent the group mean for wideband noise and narrowband noises, respectively. The hatched area and error bars represent the standard errors of the mean.

In Experiment 2, the temporal extent of PAM was examined by measuring the longest IAI for detecting the BIC embedded in either wideband or narrowband (with the CF of 200, 400, 800, 1600, or 3200 Hz) noise in the same 30 participants who participated in Experiment 1. The results showed that for the wideband noise, the longest IAI for the BIC detection varied in the range between 5.1 and 15.4 ms across 30 participants (values along the abscissa in each panel of Figure 5). For narrowband noises, individual participants’ longest IAIs generally decreased as the CF increased from 200 to 3200 Hz (values along the ordinate in each panel of Figure 5). The group-mean longest IAIs also significantly decreased as the CF increased from 200 to 3200 Hz (Figure 6) (one-way ANOVA: F(4,116) = 256.1, *p*<0.001). Obviously, this temporal extent of PAM (the longest IAI) for either wideband or narrowband noises varied remarkably across participants. Moreover, the longest IAI for the wideband noise was significantly correlated with that of each type of the narrowband noises, but the correlation coefficient generally decreased as the narrowband-noise CF increased (Figure 5).

## Experiment 3: Comparison of the Longest IAI for Detecting the BIC and the Longest IAI for Perceptually Fusing the Noises

Experiment 3 was to confirm whether the temporal extent of PAM is longer than the IAI threshold for perceptually fusing the interaurally correlated noise.

### Participants

Thirteen university students (7 females and 6 males; mean age = 23.0 yrs between 19 to 28 yrs) provided informed consent and participated in Experiment 3. They all had symmetrical hearing (no more than 15-dB difference between the two ears) and normal pure-tone hearing thresholds (no more than 25 dB HL) between 0.125 and 8 kHz. They were paid a modest stipend for their participation.

### Apparatus and Stimuli

The apparatus were the same as that used in Experiment 2. Gaussian wideband noises used in this experiment were also the same as those used in Experiment 2, except that the noise duration was only 1000 ms (including 30-ms rise-fall time) and the sound level was set at 60 dBA.

### Procedure

The design and procedure for measuring the longest IAI for detecting the BIC was similar to those used in Experiment 2.

The longest IAI for maintaining perceptual fusion was determined in a three-down-one-up procedure. In each trial, the right-ear noise always led its identical copy to the left ear. Participants were instructed to indicate whether they perceived a noise image from the left headphone by pressing the left “yes” button of the response box or nothing from the left headphone by pressing the right “no” button. In each session, the IAI was started at 72 ms and decreased after three “yes” responses and increased after one “no” response. No feedback was given to participants. The initial step size of changing the IAI was 16 ms, and the step size was altered by a factor of 0.5 with each reversal of direction until the minimum size of 1 ms was reached.

In this experiment, a test session was terminated following 10 reversals in direction and the threshold for that session was defined as the mean IAI for the last six reversals. For each participant under each condition, there were three test sessions. The mean threshold for the three test sessions was used as the longest IAI.

### Results and Discussion

**Figure 7:**
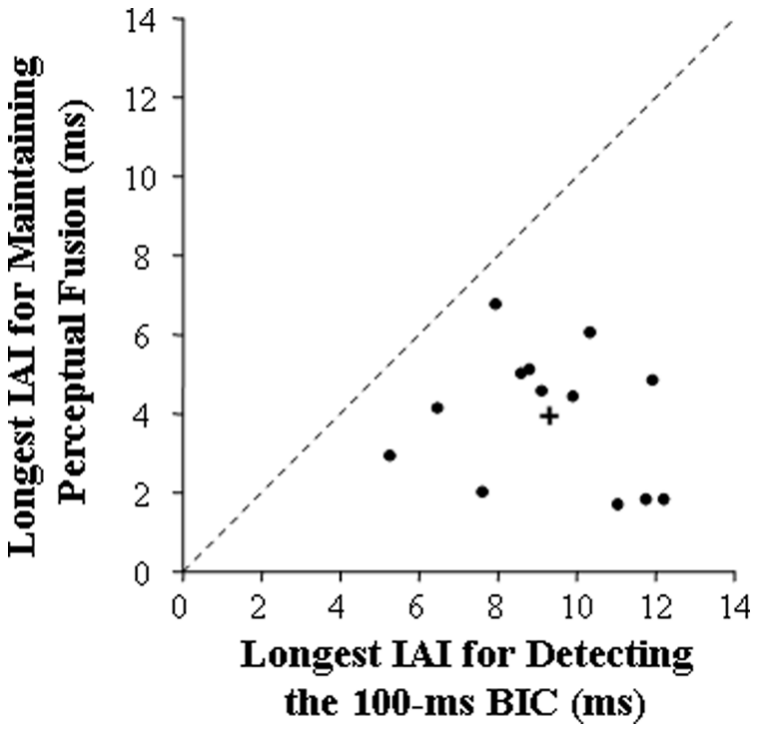
Comparison of the longest IAI when the 100-ms BIC was detectable (the abscissa) and the longest IAI when perceptual fusion of the identical noises at the two ears (the ordinate) for individual participants (dots) and group mean (the cross). The dotted straight line has the slope of 1 as a baseline for the comparison.

Figure 7 shows the longest IAI when the BIC was still detectable (the abscissa value) and the longest IAI when the perceptual fusion of the binaurally presented identical noises was still maintained (the ordinate value) for individual participants (dots) and the group mean (the cross) in Experiment 3. Clearly, for both values of individual participants and the group mean, the longest IAI for BIC detection was always larger than the longest IAI for noise fusion. A paired t-test showed that the group-mean longest IAI for BIC detection was significantly larger than that for fusion maintenance [*t* (12) = 6.41, *p*<0.001].

## Experiment 4: The BIC-Duration Threshold as the IAI Varied between 0 and 10 Ms

Experiment 4 was to estimate the dynamic features of the decay of PAM with increasing the IAI by measuring the change in the BIC-duration threshold for detecting the BIC as the IAI was varied between 0 and 10 ms. Note that with an increase of the BIC duration, it becomes easier to detect the occurrence of the BIC.

### Participants

Six participants who participated in Experiment 3 participated in Experiment 4.

### Apparatus and Stimuli

The apparatus, stimulus generation, and stimulus delivery were same to those in Experiment 3, except that the duration of BIC was systematically varied at the IAIs.

### Design and Procedures

Duration thresholds for detecting the BIC in the temporal middle of the identical (correlated) noises were tracked at each of the 6 IAIs (0, 2, 4, 6, 8, and 10 ms) using the same adaptive two-interval, two-alternative, forced-choice procedure. The BIC duration was manipulated by a three-down-one-up procedure: The duration was decreased after three consecutive correct identifications of the presentation containing the BIC and increased after one incorrect identification. For each of the IAIs, the initial duration of the BIC was sufficiently large (100 ms for the IAIs of 0, 2, and 4 ms; 300 ms for the IAIs of 6, 8, 10 ms) and the initial step size for changing the duration of BIC was 16 ms, and the step-size was altered by a factor of 0.5 with each reversal of direction until the minimum step-size of 1 ms was reached. Feedback was given visually after each trial by the LED on each button. A test session was terminated after 10 reversals in direction, and the duration threshold for a session was defined as the mean durations of the last 6 reversals. The mean duration threshold for three test sessions was used as the duration threshold for each participant.

### Results and Discussion

**Figure 8:**
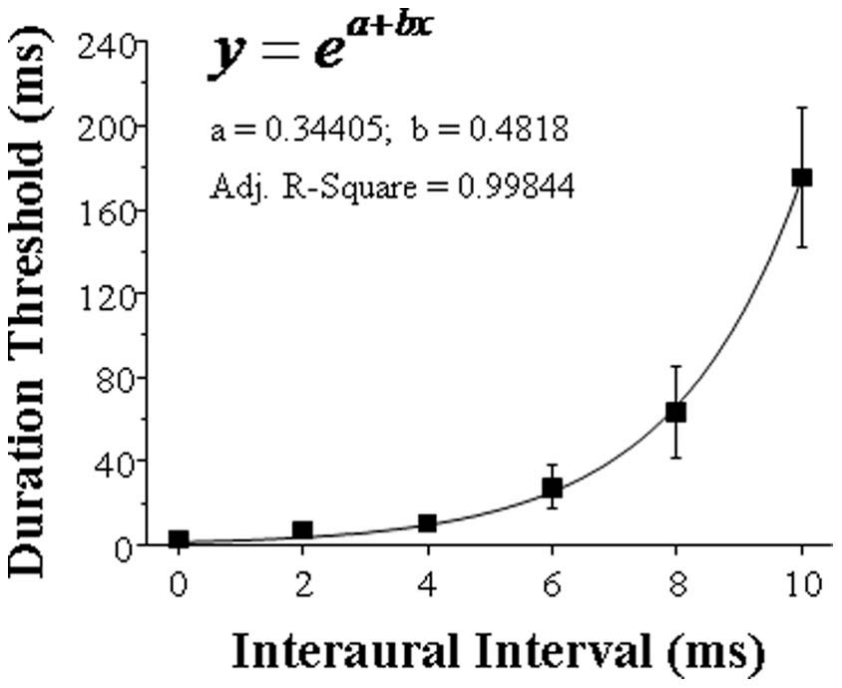
The group-mean BIC-duration threshold and the best fitting curve of mean duration threshold against IAI. **Error bars represent standard errors of the mean.**

Figure 8 shows the group-mean duration threshold for detecting the BIC as a function of the IAI for 6 participants in Experiment 4. The duration threshold increased with the increase of the IAI in accelerating fashion. A one-way within-subject ANOVA showed that the effect of IAI was significant, *F* (5, 25) = 19.535, *p*<0.001. Then the following best-fitting psychometric function was used for describing the relationship between the BIC duration threshold and the IAI (see Figure 8):
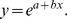
where *y* is the duration threshold for detecting the BIC when the IAI is *x*; both a and b are the constants of the function; *e* is Euler’s constant (which is 2.71828).

## Correlation between Recognition of Target Speech and the Temporal Extent of Pam

**Figure 9:**
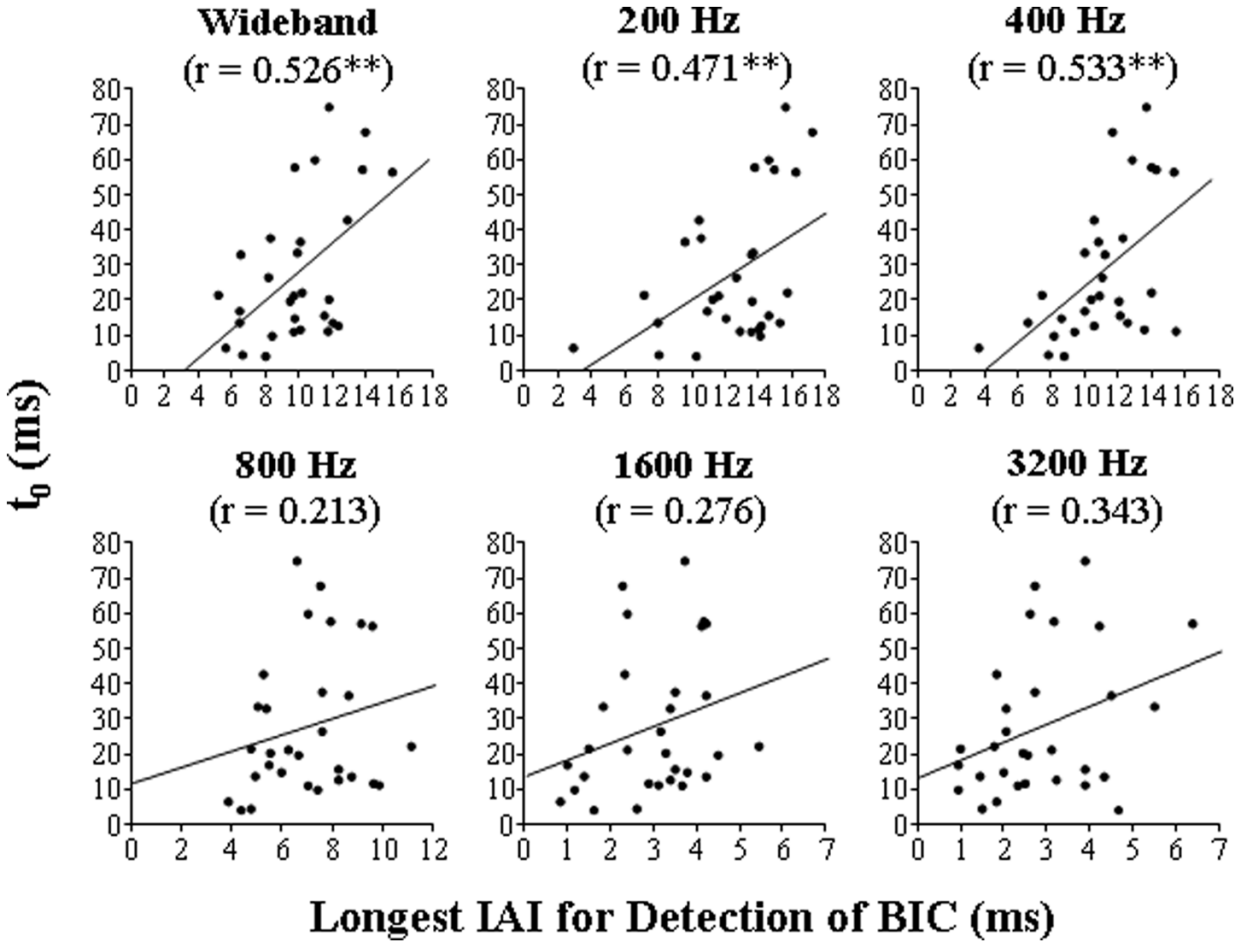
Correlation between *t*_0_ (obtained from Experiment 1) and the longest IAI for detecting the BIC in each noise type (obtained from Experiment 2) across 30 participants. In each panel, the solid line is the best linear fitting for data points and *r* is the Pearson correlation coefficient. Note that *t*_0_ was significantly correlated with the longest IAI for detecting the BIC embedded in either the wideband noise or the narrowband noise with the low CF of 200 or 400 Hz. ‘**’ indicates the significant level at 0.01.

**Figure 10:**
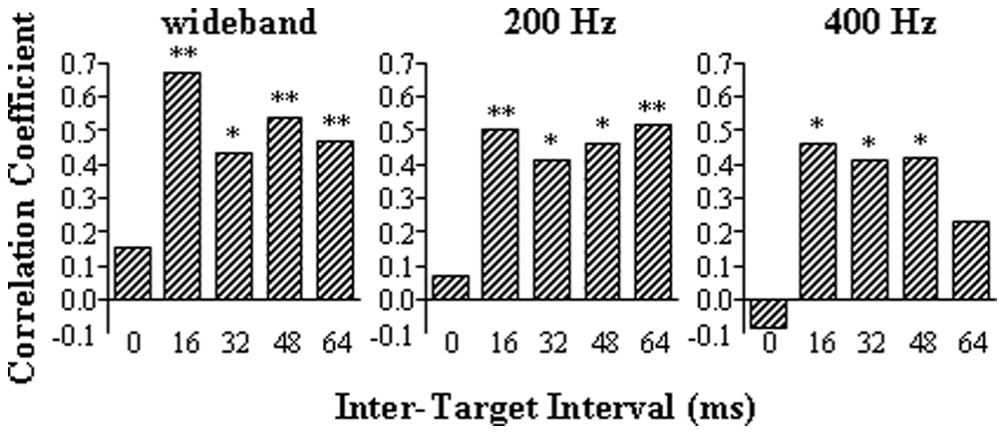
Correlations between percent-correct target-speech recognition (obtained from Experiment 1) and the longest IAI for detecting the BIC in either the wideband or the narrowband noise with the low CF of 200 or 400 Hz (obtained from Experiment 2) across 30 participants. ‘*’ indicates the significant level at 0.05; ‘**’ indicates the significant level at 0.01.

Finally, correlations were calculated between the *t*_0_ (obtained from Experiment 1) and the longest IAI for both wideband and narrowband noises (obtained from Experiment 2) across 30 participants (Figure 9). The correlation coefficient associated with each noise type is presented in the corresponding panel of Figure 9. The results indicate that the *t*_0_ was generally larger as the longest IAI became larger, but was significantly correlated with the longest IAI only for the wideband noise and the narrowband noise with the CF of 200 or 400 Hz. Moreover, for both the wideband noise and the two low-CF (200 and 400 Hz) narrowband noises, the longest IAI was significantly correlated with the target-speech recognition performance at most of the non-zero ITIs but not at the ITI of 0 ms (Figure 10).

## General Discussion

### Spatial Unmasking Based on Perceptual Integration

To improve speech recognition in noisy environments with multiple people talking, listeners use various spatial and/or non-spatial perceptual/cognitive cues to facilitate perceptual segregation of the target speech and the speech masker, largely by strengthening their selective attention to the target-speech stream [2]. Spatial separation between a target sound and its masking sounds is the most useful and reliable cue for improving recognition of the target by head-shadowing, interaural-interaction, and attentional-facilitation unmasking effects. However, when the listening environment with multiple speech sources is reverberant, numerous reflections limit or even abolish the head-shadowing and interaural-interaction effects [3]–[8], but the perceptual integration between the (leading) direct wave and the reflections of each source can cause perceived location differences between the images associated with different (uncorrelated) sources, maintaining the attentional-facilitation unmasking effect. In other words, the perceived spatial differences facilitate listeners’ selective attention to the target-speech source, leading to improvement of the target-speech recognition [4], [19]–[22], [24], [31].

The results of this study not only confirm the unmasking role of the direct-reflection perceptual integration, but also indicate that the perceptual-integration-based recognition of target speech under speech masking is a descending exponential function of the reflection delay and the temporal dynamic of this function can be represented by *t*_0_. Moreover, there is a remarkable variability across listeners in both the *t*_0_ and the range of performance that is affected by ITI (i.e., the parameter *A*).

### Features of Primitive Auditory Memory

This and previous studies [23], [24], [40]–[42] have shown that the longest IAI for detecting the BIC is far beyond the longest physiological interaural delay that is due to the difference in the direct propagation paths to the two ears for a source in the space, implying the possibility that PAM occurs at the central level [41]. Moreover, the results of this study indicate that the temporal extent of PAM (measured at the longest IAI for detecting the BIC) is even longer than the temporal threshold for binaural fusion (i.e., the longest IAI for maintaining the perceptual fusion of the interaurally correlated noises). More specifically, in Experiment 3 the group mean of the longest IAI across 13 participants for detecting the BIC was 9.3 ms, which was much larger than that of the longest IAI, 4.0 ms, for maintaining the perceptual fusion of the wideband noises (the fusion threshold). Thus, PAM can be maintained at the IAI even when the perceptual fusion of the noises at the two ears is broken. The perceptual fusion occurs only when all or most non-spatial attributes of the noise at the lagging ear are perceptually captured by the noise at the leading ear (see [10]), requiring that the IAI must be sufficiently short. However, the detection of the BIC can occur as long as the contrast between the central representation of the BIC and that of the BIC banks is sufficiently large. In other words, the detection of BIC is more tolerant to the increase of IAI than the perceptual fusion.

On the other hand, the longest IAI is much shorter than both the temporal extent of the earlier component of auditory sensory memory (up to about 200 to 300 ms) [45] and the temporal extent of the echoic memory (up to 10 s) [48]–[50]. Thus, the PAM has been suggested to be the early point of the chain of the transient auditory memory system [23]. Moreover, since processing fine-structure information is largely based on phase locking of neural firing and phase locking tends to breakdown as the frequency increases, the longest IAIs for detecting the BIC in low-frequency noise are longer than those in high-frequency noises.

As mentioned in the Introduction, the perceived size of the noise image depends on the interaural correlation (e.g., [37], [38]): At small IAIs, the noise image is compact, and a dynamic drop in interaural correlation causes a large change in size contrast; at large IAIs, the change in the size contrast becomes small. Thus, detection of changes in correlation is essentially detection of the size change of the auditory event. It is well known that detection of a small decrease in interaural correlation from a reference correlation continues to become worse as the reference correlation decreases [33], [34], [36], [51], [52]. In other words, it is much easier to detect the change from the perfectly correlated value (+1) to a slightly decorrelated value than from a slightly decorrelated value to a more decorrelated value. With increasing the IAI, the central representation of fine structure of noise is progressively diminished, and a compact, correlated image becomes broadened, leading to reduced sensitivity to changes in the interaural correlation.

In Experiment 4 of this study, the temporal dynamic of the PAM decay was estimated by investigating the change in BIC-duration threshold for detecting the BIC as the IAI varied between 0 and 10 ms. The results showed that the group-mean duration threshold increased in an exponential pattern as the IAI increased. Thus, as the IAI increases, PAM of raw signals from the leading ear rapidly decays, leading to a fast decrease in the similarity (correlation) between the central representation of the noise from the leading ear and that from the lagging ear, thereby a fast decrease in the contrast between the BIC and its banks in central representation. This study provided first evidence that PAM continues to decay in an accelerated manner, and particularly the decay becomes remarkable 8 ms after the sound arrival at the ear.

### Spatial Unmasking Based on Perceptual Integration Is Correlated to Primitive Auditory Memory

More importantly, this study reveals the functional relationship between the two types of auditory processes: One is the higher-level perceptual-integration-based recognition of target speech under speech masking and the other one is the lower-level transient storage of acoustic fine structures (i.e., the fast fading PAM), particularly low-frequency fine structures. More specifically, for the first time, this study reports that the temporal dynamic (i.e., *t*_0_) of perceptual integration-based recognition of target speech against speech masking is significantly correlated with the temporal extent of PAM (i.e., the longest IAI) for both wideband noise and narrowband noise with low CFs (200, 400 Hz).

Thus, if a listener has longer temporal extent of PAM (i.e., a stronger tolerance to IAI), exhibiting the ability to maintain low-frequency raw signals for a longer time, her/his speech recognition will have a stronger tolerance to the increase of delay of the simulated target-speech reflection (showing a slower decline in target-speech recognition as the ITI increases). Interestingly, the results of this study also showed that when the ITI was zero, listeners’ speech recognition was not correlated with the longest IAI, indicating that the function of PAM occurs only when there is a time interval between two correlated speech waves. This finding was not reported in previous studies [23], and it is opposed to the assumption that the correlations between the results of Experiments 1 and 2 were due to either higher motivation or better general performance of some listeners than other listeners, i.e. the better-listener effect.

According to the study of Goupell and Hartmann [37], in addition to interaural coherence itself, the reduction in bandwidth of narrowband noises affects the detection of incoherence by introducing both slower fluctuations and extreme values of fluctuations in both interaural-phase difference (IPD) and interaural-level difference (ILD). When the relative bandwidth (e.g., 1/3 octaves) is fixed, low-frequency noise bands have smaller absolute bandwidths than high-frequency noise bands. Although in the present study, the participants’ ability to detect fluctuations in IPD and/or ILD might affect the correlation between the results of Experiment 1 and Experiments 2, whether the change in absolute bandwidth affects the temporal extent of PAM needs further investigation.

### The Build-Up of Spatial Unmasking of Speech under Reverberation: The Chain-Processing Theory

Notionally, PAM is critical for cross-time central computation of the dynamic similarity between leading and lagging sound waves, and in turn, the central computation is critical for inducing perceptual integration between leading and lagging waves. Thus, based on the results of this study, we propose a new *Chain-Processing Theory* that explains how the spatial unmasking of target speech in reverberant environments with multiple-people talking is built up: There is an auditory signal-processing chain for recognizing speech in noisy, reverberant environments and this chain includes the following links: (1) the PAM maintaining raw acoustic signals for a period of time necessary for central computation of similarity between leading and lagging waves, (2) the cross-time central computation of the similarity (correlation) between the leading and lagging waves, (3) the perceptual integration between the leading and lagging wave signals, (4) the perceived-separation-induced facilitation of selective attention to the target speech signal, and (5) the attention-facilitation-induced improvement of recognition of target speech.

### The Final Remarks: The General Role of Perceptual Integration in Unmasking

The two eyes in humans are separated by a distance of about 6.5 cm so that each eye receives a visual image from a slightly different vantage point (the left-eye and right-eye retinal images are shifted relative to each other). The central visual system is able to use the two-dimensional binocular-disparity information to induce binocular (perceptual) integration and form three-dimensional percepts [53]–[55]. This binocular-disparity-based stereopsis is critical not only for distance judgment in depth [56], but also for unmasking the target(e.g., [54], [57], [58]). Specifically, when a target is presented stereoscopically against a disrupter, which is also presented stereoscopically, introducing a difference in binocular disparity between the target and disrupter causes perceived depth separation between the binocularly fused target image and the binocularly fused masker image(s), thereby improving the detection of the target [59]–[63].

The existence of reflections in the environment complicates the scene analysis for speech recognition to such an extent that no currently existing computer system, which lacks perceptual integration, is capable of accomplishing this task. The perceptual integration-based spatial unmasking of auditory signals shares the basic principle of perceptual integration with the binocular unmasking [60], [62]. Thus, the perceptual integration is the strategy generally for untangling complex perceptual scenes.

In conclusion, investigation of how speech is recognized in a noisy, reverberant environment is useful for understanding the basic principles of how the human brain extracts meaningful information against sensory ‘flooding’, the enormous input of sensory signals from various sources.
